# Sleep, napping and alertness during an overwintering mission at Belgrano II Argentine Antarctic station

**DOI:** 10.1038/s41598-019-46900-7

**Published:** 2019-07-26

**Authors:** Agustín Folgueira, Guido Simonelli, Santiago Plano, Camila Tortello, Juan Manuel Cuiuli, Abel Blanchard, Alejandro Patagua, Allison J. Brager, Vincent F. Capaldi, André E. Aubert, Marta Barbarito, Diego A. Golombek, Daniel E. Vigo

**Affiliations:** 1 Neurology Department, Central Military Hospital, Argentine Army, Buenos Aires, Argentina; 2 Behavioral Biology Branch, Walter Reed Army Institute of Research, Silver Spring, Maryland USA; 3 Argentine Joint Antarctic Command, Buenos Aires, Argentina; 4 Chronobiology Lab, National University of Quilmes (UNQ), Argentina and National Scientific and Technical Research Council (CONICET), Buenos Aires, Argentina; 5 Argentine Antarctic Institute, Buenos Aires, Argentina; 6 Faculty of Psychology and Educational Sciences, Katholieke Universiteit Leuven, Leuven, Belgium; 7 Chronophysiology Lab, Institute for Biomedical Research (BIOMED), Catholic University of Argentina (UCA) and National Scientific and Technical Research Council (CONICET), Buenos Aires, Argentina

**Keywords:** Circadian rhythms and sleep, Attention, Epidemiology

## Abstract

During Antarctic isolation personnel are exposed to extreme photoperiods. A frequent observation is a sleep onset phase delay during winter. It is not known if, as a result, daytime sleeping in the form of naps increases. We sought to assess sleep patterns - with focus on daytime sleeping - and alertness in a Latin American crew overwintering in Argentine Antarctic station Belgrano II. Measurements were collected in 13 males during March, May, July, September and November, and included actigraphy and psychomotor vigilance tasks. Sleep duration significantly decreased during winter. A total of eight participants took at least one weekly nap across all measurement points. During winter, the nap onset was delayed, its duration increased and its efficiency improved. We observed a significant effect of seasonality in the association of evening alertness with sleep onset. Our results replicate previous findings regarding sleep during overwintering in Antarctica, adding the description of the role of napping and the report of a possible modulatory effect of seasonality in the relation between sleep and alertness. Napping should be considered as an important factor in the scheduling of activities of multicultural crews that participate in Antarctica.

## Introduction

Light of sufficient intensity and adequate spectral composition is one of the main factors that maintain the 24-hour period of human circadian rhythms. Therefore, sleep as a bio-behavioral state is heavily shaped by our biology and by environmental factors. In high latitudes such as the ones found in Antarctica, personnel are exposed to extreme photoperiods. Probably one of the greatest concerns during Antarctic isolation is sleep disruption. A frequent observation is a sleep onset and a circadian temperature rhythm phase delay during winter, or even completely desynchronized or free-running patterns. A delay of circadian rhythm superimposed on the normal working hours implies that sleep is attempted at a suboptimal phase. During the winter, there are also decreases in efficiency, latency, duration and quality of sleep^1,2^.

Sleep is a major determinant of alertness, but there are few reports about changes in performance during Antarctic missions. For example, one study showed performance decrements during a four-month summer expedition^3^. In a study that used a computerized simulation of a complex life support system, no signs of serious performance decrements were observed, but subtle indications of hidden decays appeared^4^. A recent study at Halley Station evaluated the effect of light pulses of one hour for periods of two weeks, and their intervention resulted in a circadian phase advance and improved objective cognitive performance with no changes in  subjective alertness^5^.

In this context, daytime sleeping in the form of naps may increase^6,7^. Napping is a cross-cultural phenomenon for which its prevalence ranges between different countries from 36% to 80%^8^. Napping may be a response to or in preparation to sleep loss. It is also considered as a countermeasure to sleepiness in shift workers or people with other sleep disorders. Usually, it is associated with improvements in alertness, mood and performance^9^. In addition, napping could be part of the cultural and societal factors that also shape individual’s sleep. Illustrative of this are the siesta cultures, often seen in equatorial climates where post-prandial naps are common. Other cultural differences that may be related to this habit include the well-described disparities between southern and northern European countries in terms of meal timing and, consequently, bed timing^10^.

However, we have not found studies that reported daytime sleeping during over-wintering missions in Antarctica stations nor its possible role in the modulation of alertness variations. Belgrano II Argentine Antarctic station is at a similar distance from the pole to other stations where behavioral studies are often performed, such as Halley VI (United Kingdom) and Concordia (France – Italy), sharing with these stations similar photo-periodicities^11–13^. Siesta is socially acceptable and encouraged in many parts of Argentina, and this is perhaps better reflected by the extent to which it is common in many settings to have a long break after lunch, compatible with siesta practice^14,15^. The work schedule (and routine) for Belgrano II station is not an exception to common Argentine sleep practices, and crewmembers have a 90-minute break after lunch, that individuals can choose to use for a siesta. Therefore, this Antarctic station provides a new and different scenario and an excellent model to study sleep health and sleep patterns in the context of an extreme environment.

There are relevant reasons why studying sleep patterns in different cultures in these settings are important. First, in the context of space exploration, Antarctica is often seen as one of the best space analogues to study human adaptation to confinement and to a hostile environment^16^. For example, this has specific relevance for the design of a future manned spaceflight to Mars. Testing different sleep patterns in these contexts may help identified protective sleep practices from a health and human performance standpoint. Second, as higher latitudes become more habitable due to global warming, understanding how different cultures adapt to extreme photoperiods and environments may help to identify strategies to mitigate the effect of an adverse environment and facilitate successful human adaptation. Third, it may help elucidate the extent to which sleep practices are dictated by the environment versus the society. Existing data on Antarctic sleep patterns has focused primarily in individuals from non-siesta cultures. Finally, extreme environments and specifically extreme photoperiods, might provide a mechanistic insight into pathophysiological processes associated with sleep disruptions. This could be relevant in the search of early circadian biomarkers of disorders like mild cognitive impairment and Alzheimer’s disease^17–19^.

Thus, the main objective of this study was to assess sleep patterns and alertness in a Latin American crew during a one year overwintering mission in Antarctica. As a secondary objective, we sought to explore whether sleep-wake cycle during the year modulate possible alertness variations.

## Results

### Clinical data

A total of 13 male army personnel were included in the study, with a mean age of 34 ± 1 years. Initial body mass index (BMI) was 26 ± 1 kg/m^2^, showing a mild overweight pattern. Initial systolic and diastolic blood pressure values were 113 ± 2 / 66 ± 1 mmHg, both within normal values. BMI and blood pressure did not present significant variations during the year.

### Sleep

**Figure 1:**
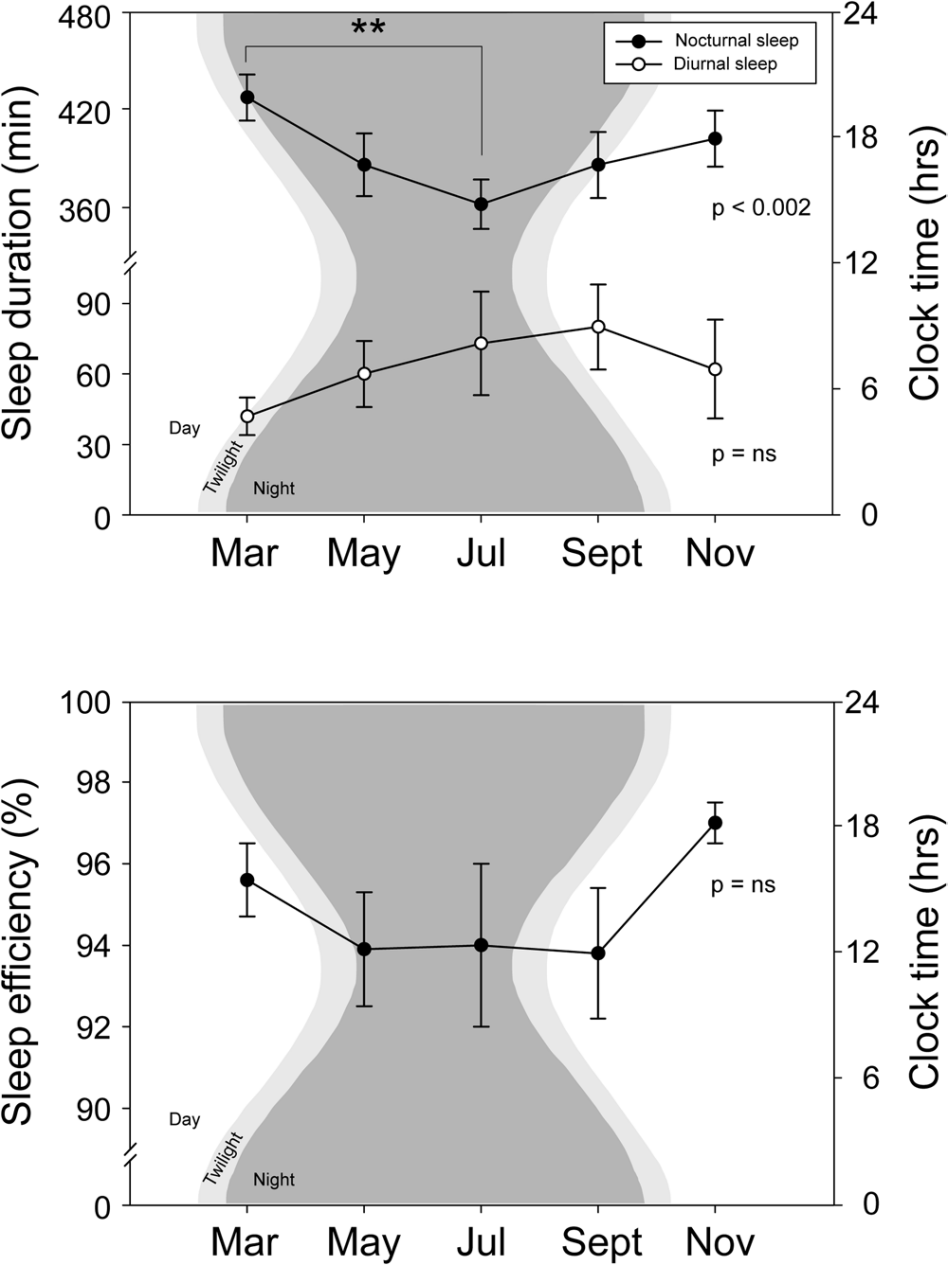
Top panel: Nocturnal and diurnal sleep at Belgrano II Antarctic station. Nocturnal sleep duration decreased in July. The average daytime sleep duration and the ratio between diurnal and nocturnal sleep for the crew showed a non – significant increase during winter. Bottom panel: Nocturnal sleep efficiency. Sleep quality during nocturnal sleep showed non-significant changes throughout the year. For both panels, shown are means ± SEM. Statistical differences assessed by repeated measures ANOVA followed by Bonferroni post-hoc tests (**p < 0.01). In the background, panels show the duration of natural sunlight (daylight + civil twilight) and night periods (https://www.timeanddate.com/sun/antarctica/belgrano-ii-base).

Table 1 shows actigraphic sleep outcomes at each measurement point. Data from one individual was excluded due to an incomplete recording caused by a malfunctioning actigraph, resulting in a final sample of 12 individuals. Mean sleep duration significantly decreased during the polar night (July) (p < 0.002, Fig. 1), while a later sleep onset was observed during the same period, although not statistically significant (p < 0.051). Sleep offset and sleep efficiency (Fig. 1) did not show significant changes throughout the year.

**Table 1:** Sleep characteristics.

	Mar		May		July		Sept		Nov		p
	Mean	SEM	Mean	SEM	Mean	SEM	Mean	SEM	Mean	SEM	
***Nocturnal sleep***											
Recording length (number of nights)	6.8	0.3	6.4	0.5	6.0	0.3	6.3	0.4	5.9	0.4	0.600
Onset (HH:MM)	01:01	:14	01:11	:24	01:55	:16	01:43	:19	01:34	:18	0.051
Offset (HH:MM)	08:08	:07	07:37	:12	07:58	:12	08:10	:14	08:16	:13	0.085
Duration (min) (1)	427	14	386	19	362	15	386	20	402	17	0.002
Efficiency (%)	95.6	0.9	93.9	1.4	94.0	2.0	93.8	1.6	97.0	0.5	0.352
Diurnal sleep*											
**Daily averages**											
Number of sleep episodes (weekly average)	3.0	0.5	3.6	0.8	3.5	0.7	3.4	0.6	3.2	0.9	0.856
Duration (min)	42	8	60	14	73	22	80	18	62	21	0.366
Diurnal/Nocturnal	0.10	0.02	0.16	0.04	0.20	0.06	0.20	0.05	0.15	0.05	0.305
**Sleep episodes averages**											
Onset (HH:MM) (2)	14:28	:15	15:43	:21	15:38	:12	15:23	:32	15:41	:40	0.029
Offset (HH:MM) (3)	16:12	:20	17:44	:25	18:14	:21	18:07	:21	17:44	:41	0.002
Duration (episode average, min) (4)	104	8	121	12	157	15	164	28	122	13	0.006
Efficiency (%) (5)	88.1	1.8	93.3	1.3	93.9	1.8	94.6	2.0	95.6	0.7	0.016

^*^For diurnal sleep, the daily averages were calculated averaging over the length of the measurement period (i.e. including days with and without diurnal sleep). The sleep episodes averages were calculated averaging over the number of the nap episodes of the measurement period (i.e. including only those subjects who slept at least one nap during each measurement period, n = 8). (1) July < Mar; (2) July > Mar; (3) July & Sept > Mar; (4) July > Nov; (5) post-hoc: ns. Repeated measures ANOVA followed by post-hoc Bonferroni test.

**Figure 2:**
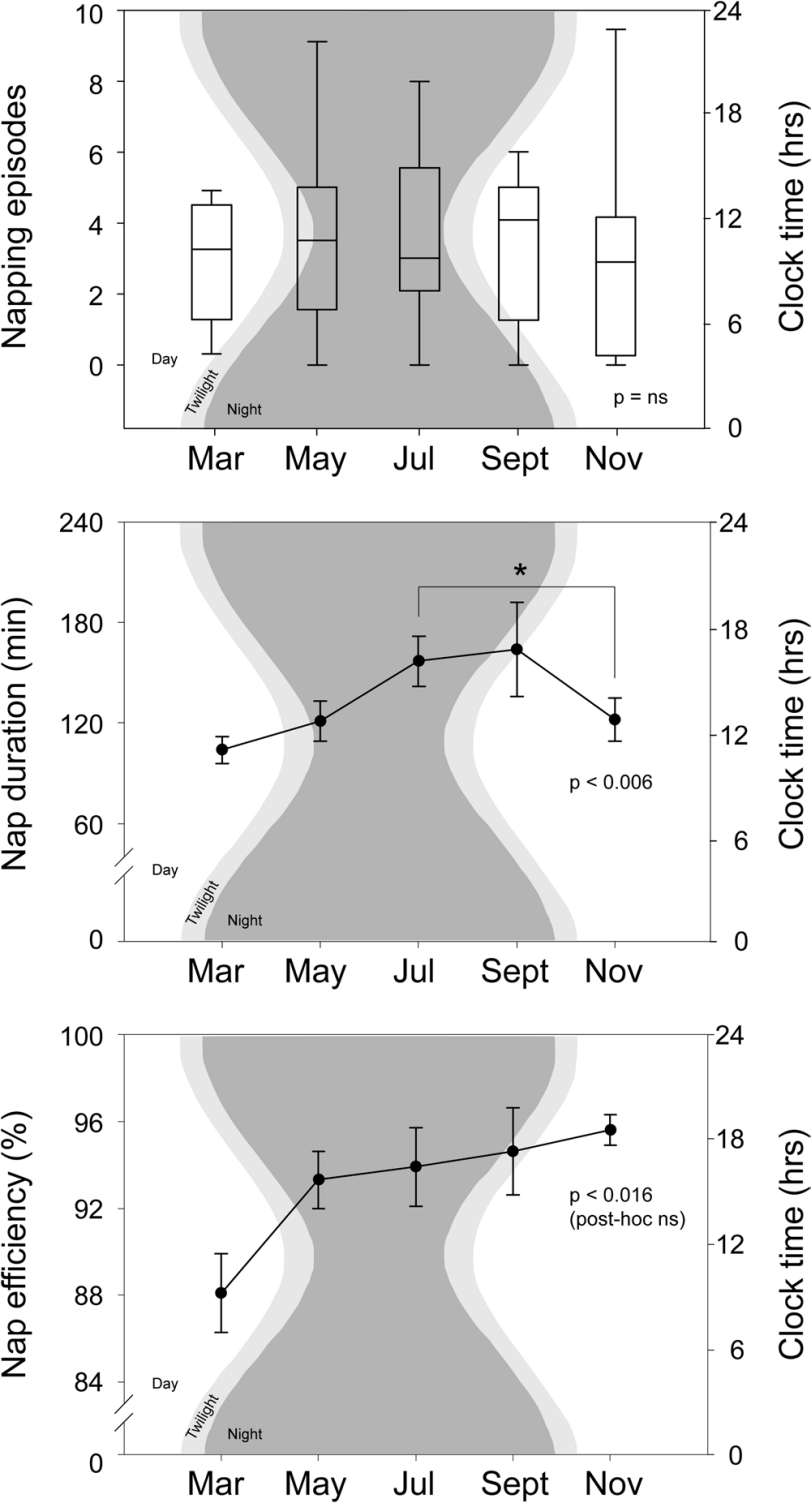
Napping episodes. Top panel: Distribution of napping episodes along the year. Individuals took around three naps per week. Shown are medians, 25–75 and 10–90 percentiles. Mid panel: Nap duration. A minimum of nap duration is observed in July. Shown are means ± SEM. Bottom panel: Nap efficiency. Sleep quality during naps increased significantly after March and maintained similar mean values thereafter. Shown are means ± SEM. For all panels, statistical differences assessed by repeated measures ANOVA followed by Bonferroni post-hoc tests (*p < 0.05). Napping duration and napping efficiency averages were calculated only for crewmembers who took at least one nap during each measurement period. In the background, panels show the duration of natural sunlight (daylight + civil twilight) and night periods (https://www.timeanddate.com/sun/antarctica/belgrano-ii-base).

In terms of napping, the average daytime sleep duration and the ratio between diurnal and nocturnal sleep for the crew, duplicated their values in July when compared with March, although these differences were not statistically significant (Fig. 1). The participants took around three weekly naps during each measurement point (Fig. 2). Interestingly, the nap episode itself (i.e. not the daily diurnal sleep average) which was calculated only for those individuals who took at least one nap during each measurement period (rather than the whole crew), increased significantly its duration with a maximum of 164 ± 28 min in September (Fig. 2), and delayed significantly its onset (with a significant maximum in July) and offset (with significant maxima in July and September). The nap’s sleep efficiency also showed significant changes (post hoc analysis non significant, Fig. 2).

Regarding self reported sleep quality, three data points of the PSQI questionnaires (from different participants and measurement points) and one of ESS were not available and therefore were not included in the analysis. The PSQI and the ESS both showed normal mean values during the whole year, with no significant variations.

### Alertness

Table 2 shows the morning and evening values derived from the administration of the alertness PVT. None of the analyzed variables showed significant changes throughout the year.

**Table 2:** Alertness

	Mar		May		July		Sept		Nov		p
	Mean	SEM	Mean	SEM	Mean	SEM	Mean	SEM	Mean	SEM	
***Morning***											
MRT (ms)	284	8	290	15	284	11	282	11	286	13	0.470
SRT (ms)	83	10	129	22	111	23	93	18	94	19	0.917
FRT (ms)	209	5	205	6	204	4	205	4	206	6	0.161
IRT (1/ms)	2.36	0.08	2.27	0.11	2.48	0.17	2.45	0.15	2.36	0.17	0.839
LRT (%)	2.2	0.5	4.6	1.9	2.5	0.8	2.4	0.8	3.0	0.9	0.615
***Evening***											
MRT (ms)	294	13	291	16	305	21	292	12	301	20	0.869
SRT (ms)	130	25	124	26	107	26	92	19	112	25	0.325
FRT (ms)	209	8	203	5	209	6	210	5	210	8	0.382
IRT (1/ms)	2.24	0.13	2.38	0.19	2.28	0.21	2.39	0.14	2.35	0.22	0.601
LRT (%)	3.6	0.8	3.4	1.4	4.5	2.0	2.3	0.8	4.9	2.2	0.805

MRT: mean response times for all trials; SRT: standard deviation of the response times for all trials; FRT: fastest 10% of response times for all trials; IRT: slowest 10% of reciprocal response times for all trials; LRT: percentage of response times ≥ 500 ms for all trials. All variables showed non-significant differences between measurements. Repeated measures ANOVA followed by post-hoc Bonferroni test.

### Association between sleep and alertness

Lastly, we found no significant correlations when testing the associations of sleep with alertness on each measurement point. Results from linear mixed model analyses, however, revealed a significant effect of time of the year in the association of morning MRT with average napping duration (p < 0.040) and in the association of evening MRT with sleep onset (p < 0.004) and % rhythm (p < 0.001).

## Discussion

The main finding of this study is that crewmembers slept on average an hour less during July (polar winter) as compared to March, and, to a lesser extent, November. Although not statistically significant, we also found suggestive evidence of a delay in sleep onset during this season. For the first time in an Antarctic crew, our results also showed that more than half of the participants chose to take at least one weekly nap, and during the winter, the onset of the nap was delayed, its duration increased and its efficiency improved. Interestingly, changes in nap duration and timing did not account for the difference observed in the 24 h sleep duration between summer and winter. We did not observe differences in PVT performance throughout the year, but we found evidence of seasonality having a modulatory role in the relation of sleep with alertness.

As for the physiological variables recorded in our sample, BMI was slightly higher than normal and blood pressure values were relatively low, with no significant variations during the year. These figures could be explained by the fact that our sample was mainly comprised by healthy and physically fit army personnel who, as part of their army duties, undergo extensive physical training. In these kinds of populations, blood pressure values may be low due to regular physical aerobic exercise^20^ and BMI values may be high due to increased free-fat muscular mass^21^.

Our work is the first one to report on sleep from a Latin American crew overwintering in Antarctica. Previous work in Antarctica has mainly studied European, Asian, North American or Oceanian crews^1,2^. To our knowledge, only one study reported on sleep in Antarctica from a Latin American crew (Uruguay), although this was during a summer campaign and in an Antarctic station located at a much lower latitude than Belgrano II, approximately 2,000 km north^22^. In our study, we found a decrease in sleep duration possibly associated with a phase delay of sleep onset during winter, consistent with the absence of natural bright light during July. In March (17:36 hrs of sunlight) and November (24:00 hrs of sunlight) the use of blinds, and eye masks could prevent the forced phase shift caused by increased light exposure^2^. The use of preset morning alarms to respect the fixed working schedule would explain why no seasonal differences were found in sleep offset. We did not observe decreases in sleep efficiency although they have been reported in the literature^1,2^.

The current study is also the first to report on napping in an isolated and confined population in an extreme environment. Throughout the mission, those who chose to take weekly naps continued to do so. On average, naps increased slightly in duration and efficiency, suggesting an increased sleep pressure. It is also not clear whether naps were prolonging as a consequence of shorter sleep at night, or vice versa. The increase in napping duration would be consistent with previous findings from a prolonged confinement study that simulated a 520-day Mars mission (Mars 500), where it was reported increased sleep and rest times as well as decreased active wakefulness time, leading to increased lethargy^7^. It would be also consistent with autonomic activity changes observed in Mars500 crewmembers, where parasympathetic activity, as measured by heart rate variability, increased during daytime^23,24^. Further, our results highlight the role of culture shaping our sleep health. In our study, the majority of the participants took naps^25^, whereas in Mars 500, only one participant chose to do so^7^. Napping has been shown to be an effective countermeasure to improve performance^25^, although sometimes stigmatized^26^.

We found no impairments in performance throughout the year using the 5-minute PVT. As previously described in the introduction, there are few reports on alertness modifications during Antarctic stays, with conflicting results. In addition, a correlation between sleep and circadian rhythms with alertness could not be established. Probably, the decrease in sleep duration may not have been sufficient to determine changes in sustained attention as measured by PVT. However, we did find a modulatory role of seasonality in the correlation of sleep onset and phase angle with mean reaction time. It is possible that the impact of sleep features in alertness could be different depending on the moment of the year, as it was shown with the exposure to bright light during the morning, which significantly improved alertness during the dark months of the year, but not during spring^27^.

Our study has a number of strengths. Belgrano II is located at extremely high latitude (comparable to the one of Concordia or Halley VI), relatively close to the South Pole and isolated. Belgrano II is also at sea level, removing the potential impact of hypoxia as a confounding factor found in, for example, Concordia. Also, measurements were collected during most of the year, allowing the comparison of different photoperiods. Further, in our study we included not only sleep measures but also performance metrics, leading to a more comprehensive and broader assessment of individuals’ sleep health.

Some limitations should be mentioned as well. First, we did not include urban controls (pre/post), although the aforementioned Uruguayan study found no significant differences between those measurements^22^. In addition, measurements were not conducted during December or February because in the period after arrival and before departure from Antarctica, crewmembers were exposed to intensive working schemes related with the logistics of crew change. Therefore, measurements started 1.5 months after arrival and ended 1.5 months before departure. Another limitation of the study is the inclusion of only young males, which may decrease generalizability. For example, it was reported that women (but not men) showed declining sleep quality and men (but not women) showed declining physical activity during over wintering in Antarctica, attributed to a different susceptibility to social or environmental factors^28^. To our knowledge, no studies have assessed sleep patterns in older adults in Antarctica. Lastly, we only included one week of actigraphy per month, per individual, due to logistical constrains. The inclusion of one week of actigraphy though, is often considered as sufficient to characterize someone’s sleep^29–31^.

As already stated, this is the first study to describe sleep patterns in a Latin American crew during an overwintering Antarctic mission. To some extent, our results replicate previous findings regarding sleep disruption during overwintering in Antarctica, documenting for the first time a possible modulatory effect of seasonality in the relation between sleep and alertness, and a description of napping in these extreme conditions of isolation and confinement. Either as a cross-cultural phenomenon or as a result of its benefits in alertness, mood and performance, napping should be considered as an important factor in the scheduling of activities of multicultural crews that participate in Antarctica or international space missions.

## Methods

### Participants and design

Argentine Antarctic research station Belgrano II is constituted by a series of scientific research facilities located at sea level on the mainland at the Nunatak Bertrab, in front of the Weddell Sea (77° 51′S and 34° 33′W). It is located approximately 1,300 km from the South Pole, being one of the southernmost permanent stations on the planet. The crew of the station usually consists of 18 men, 13 Argentine Army personnel, two members of the National Weather Service, and three scientists of the Argentine Antarctic Institute.

Due to its geographical location, Belgrano II has around four months (from October 22^nd^ to February 18^th^) of day (polar day: at least part of the Sun’s disk is visible during 24 hrs), four months (from February 19^th^ to April 23^rd^ and from August 19^th^ to October 21^st^) of variable day lengths and four months (from April 24^th^ to August 18^th^) of night (polar night: none of the Sun’s disk is visible during 24 hrs) (dates correspond to the year 2014). The temperature ranges between 5 °C in summer and −43 °C in winter, with gusts of wind above 200 km/h. To generate a light-dark cycle during the summer, windows with closed blinds are used during the “night”, agreeing on a normal sleep routine and using eye covers if necessary. Exposure to ultraviolet light is also stronger and the personnel of the stations require solar glasses for external work. During the winter, however, the light-dark cycle relies entirely on artificial lighting. The bedrooms and common areas were artificially lit by warm light fluorescent tubes (Phillips TLD 36 W / 830; 3250 lm, 3000 K), while the medical room was artificially lit by cool daylight fluorescent tubes (Phillips TLD 36 W / 54; 2500 lm, 6200 K). The estimated illuminance was 170 lx to 330 lx in bedrooms, 290 lx in common areas and 510 lx in the medical room.

A day organized with 24 h schedule that includes periods of work, feeding and rest is essential in Antarctic stations. A regular workday consisted of a 9:00 a.m. to 6:30 p.m. schedule with a 90-minute break in the afternoon. Similarly, strict timing for breakfast, lunch and dinner, are enforced as much as possible by the command structure. Because of the extremely low temperatures and total darkness during the Antarctic winter, Belgrano II is considered the most isolated Argentine station. For example, in the winter, emergency rescue missions by ship or plane are not feasible. The crew arrives at the research station in a research vessel along with sufficient supplies for the upcoming year, and therefore neither rationing nor caloric intake restriction is needed during the mission. Finally, the crew stays in communication with the outer world via internet and a satellite phone communication system^32^.

A total of 13 male army military personnel from the Argentine Antarctic station Belgrano II were invited to participate in this observational, analytical and longitudinal study. All participants were healthy, according to medical histories and physical examinations performed before their selection as crewmembers. The Antarctica mission began in mid-January 2014 and ended in mid-January 2015. Measurements were collected every other month from March to November. The natural sunlight period (daylight + civil twilight) duration on the 15^th^ day of each month was: March, 17:32 hrs; May, 00:00 hrs; July, 00:00 hrs; September, 14:01 hrs; and November, 24:00 hrs (https://www.timeanddate.com/sun/antarctica/belgrano-ii-base).

The study was approved by the Ethics Committee from Universidad Nacional de Quilmes (Argentina) and was performed in accordance with the Declaration of Helsinki and its amendments. Participants were informed about the nature and purpose of the study, and then invited to participate in the study. All the participants provided written informed consent.

### Measures

#### Clinical data

BMI was calculated from measured values of height and weight. Blood pressure is reported as the weekly average of the daily determinations obtained through a validated automated blood pressure oscillometric device (Omron HEM-7113, Shanghai, China) after breakfast, following the American Heart Association recommendations^33^.

#### Sleep questionnaires

The Pittsburg Sleep Quality Index (PSQI) and the Epworth Sleepiness Scale (ESS) were administered to assess sleep quality and daytime sleepiness. A cut-off of > 4 points was considered indicative of poor sleep quality in the PSQI and a cut-off of > 10 points was considered indicative of excessive daytime sleepiness in the ESS. Both questionnaires have been previously validated in Spanish^34,35^ and were administered once at each measurement time-point with the intention of capturing aspects of the sleep experience from the previous month.

#### Actigraphy and sleep logs

Sleep was assessed by wrist accelerometers (MicroMini Motionloggers Actigraphs, Ambulatory Monitoring Inc., Ardsley, NY). Participants were asked to wear the devices during seven days on the non-dominant wrist at each measurement time-point. The recordings were analyzed by the software provided by the manufacturer (Action-W User’s Guide, Version 2.4; Ambulatory Monitoring, Inc., Ardsley, NY). In addition, participants completed a sleep log with information on their main sleep period and naps. The following indexes were reported: number of recorded nights, sleep onset (starting time of the first sleep episode after bedtime, as recorded by actigraphy), sleep offset (ending time of the last sleep episode before waking-up time, as recorded by actigraphy), sleep duration (time elapsed between onset and offset), sleep efficiency (ratio expressed as % of the total time spent sleeping compared to the total amount of time spent in bed). Sleep onset values were linearized before statistical analyses were conducted. For daytime sleep episodes, the mean number of sleep episodes observed during the measurement period, and the mean daytime sleep duration were calculated averaging by the length of the measurement period, thus including days with and without daytime sleep. We also reported the ratio of daytime sleep compared to the nighttime sleep. Conversely, the nap mean onset, offset, duration and efficiency were calculated averaging by the number nap episodes, for those participants who slept at least one nap at each measurement period^29–31^.

#### Psychomotor vigilance task

We used a netbook-based adapted version of the Walter Reed Army Institute of Research five-minutes palm-held psychomotor vigilance task (PVT) to assess reaction time as a proxy of alertness. The PVT was administered in the early morning after breakfast and in the late afternoon before dinner, on one  day for each of the measuring time points. Five different indexes were reported: the mean response times for all trials (MRT); the standard deviation of the response times for all trials (SRT); the fastest 10% of response times for all trials, or optimum response domain (FRT); the slowest 10% of reciprocal response times for all trials, or lapse domain (IRT); and the percentage of response times ≥500 ms for all trials, or percentage of lapses (LRT)^36,37^.

### Statistical analysis

Values are reported as mean ± SEM. Differences between measurement points (March, May, July, September and November) were assessed by means of repeated measures ANOVA. An uncorrected F test was reported when sphericity was assumed, which was tested with Mauchly’s test of Sphericity. A  Greenhouse – Geisser correction was presented otherwise. Multiple comparisons between measurements points were conducted by means of a Bonferroni post-hoc test.

Bivariate correlations of mean reaction time with sleep onset, sleep duration, sleep efficiency, diurnal sleep average and average napping duration were explored through Pearson correlation tests, adjusted for multiple comparisons by the Bonferroni procedure. Then, to test whether sleep-wake cycle during the year (i.e. the interaction between sleep-wake cycle and seasonality) modulate alertness variations, a linear mixed model analysis was conducted. Mean reaction time was included as a dependent variable, measurement month as a repeated fixed factor, and the aforementioned indexes as covariates in separate models.

Data was tested for normality using the one- sample Kolmogorov–Smirnov test. All reported tests were performed two-tailed, with an alpha value set at 0.05.

## Data Availability

The datasets analyzed during the current study are available from the corresponding author on reasonable request.
